# Influence of *ADRB2* variants on bronchodilator response and asthma control in a mixed population

**DOI:** 10.36416/1806-3756/e20250066

**Published:** 2025-07-31

**Authors:** Pedro Augusto Silva dos Santos Rodrigues, Álvaro Augusto Souza da Cruz, Helena Mariana Pitangueira Teixeira, Luciano Gama da Silva Gomes, Hatilla dos Santos Silva, Juliana Lopes Rodrigues, Almirane Lima de Oliveira, Cinthia Vila Nova Santana, Gabriela Pimentel Pinheiro das Chagas, Camila Alexandrina Viana de Figueiredo, Ryan dos Santos Costa

**Affiliations:** 1. Departamento de Biorregulação, Laboratório de Imunofarmacologia e Biologia Molecular, Instituto de Ciências da Saúde, Universidade Federal da Bahia, Salvador (BA) Brasil.; 2. Fundação ProAR, Universidade Federal da Bahia, Salvador (BA) Brasil.

**Keywords:** Bronchodilator agents, Beta-2 adrenergic receptor, Genetic variation;, Asthma, Ethnic groups

## Abstract

**Objective:**

Given that b_2_ agonists constitute the primary treatment for asthma and that treatment response varies as a result of polymorphisms in the ADRB2 gene, we sought to investigate the associations between ADRB2 gene variants and bronchodilator response (BDR) in asthma patients.

**Methods:**

A genetic database comprising 813 individuals was analyzed for variants in the ADRB2 gene. A longitudinal analysis of severe asthma patients was performed to evaluate changes in BDR over time.

**Results:**

The rs1042713, rs1042714, and rs1042717 variants were associated with age-related changes in BDR in patients with severe asthma. The G allele (rs1042714) and the A allele (rs1042717) were associated with uncontrolled asthma, with carriers of the G46/G79/A252 alleles showing a higher risk of difficult-to-control asthma. Notably, no association was found between these variants and ADRB2 expression levels.

**Conclusions:**

Our findings suggest that a genetic panel including ADRB2 variants, as well as age-related differences in BDR, is a useful complementary tool in asthma management.

## INTRODUCTION

Asthma is a chronic disease that affects more than 339.4 million individuals worldwide^1^ and approximately 20 million people in Brazil.^2^ The main features of asthma are lower airway respiratory symptoms associated with recurrent and typically local inflammation and airflow limitation that can be reversed with the use of a bronchodilator.^3^ ^,^ ^4^

The goal of asthma treatment is to control asthma symptoms and prevent disease progression with the use of inhaled corticosteroids (ICS) in combination with long-acting β_2_ agonists (LABAs) and short-acting β_2_ agonists (SABAs).^5^ ^,^ ^6^ SABAs are also used in spirometry, which is useful for diagnosing, monitoring, and assessing asthma severity and treatment effectiveness.^2^ The bronchodilator action of β_2_ agonists occurs through the activation of the β_2_-adrenergic receptor coupled to a G protein, which stimulates the adenylate cyclase and cyclic adenosine 3’,5’-monophosphate pathways, promoting airway smooth muscle relaxation.^7^

Approximately 39% of asthma patients have poor disease control, persistent symptoms, and exacerbations, which may be associated with the individual genetic background.^8^ Inadequate clinical control and decreased bronchodilation (tachyphylaxis) have both been linked to the chronic use of β_2_-adrenergic bronchodilators.^9^ Immediate removal of agonists may promote recycling of receptors back to membrane; however, persistent activation can lead to degradation and reduced synthesis of new receptors.^10^ Furthermore, studies indicate that aging is associated not only with a reduced acute response to bronchodilators but also with a chronic loss of reversibility.^11^

Single nucleotide polymorphisms (SNPs) are the main types of genetic variations associated with changes in bronchodilator response (BDR) phenotypes in individuals with asthma,^8^ which can lead to altered structure of proteins and the rate of protein or gene expression, contributing to a variety of responses to treatments.^12^ Polymorphisms of the *ADRB2* gene, which encodes the β_2_-adrenergic receptor, are related to changes in function and coupling of agonists, particularly the rs1042713 (G46A) and rs1042714 (G79C) variants, which result in the alteration of the amino acid glycine at codon 16 to arginine and from the glutamic acid at codon 27 to glutamine, respectively.^13^

Such variations have been associated with changes in respiratory function and response to β_2_ agonists in previous studies in Brazil and worldwide.^14^ ^,^ ^15^ However, there are divergences in the literature regarding the effect of *ADRB2* polymorphisms on BDR.^16^ The conclusions of previous studies may have been influenced by variables such as the number of participants and heterogeneity of populations, highlighting the need for further studies. Therefore, the present study sought to assess the influence of *ADRB2* gene variants on the response to asthma treatment in a sample of urban adults in Brazil. 

## METHODS

### Characterization of the study population

The present study included 401 individuals who had a diagnosis of severe asthma^4^ as confirmed by two experts and who were followed under the auspices of the *Programa para o Controle da Asma na Bahia* (ProAR, Bahia State Program for the Control of Asthma). All 401 patients were classified as having severe asthma in accordance with international criteria, as described in detail elsewhere.^3^ An additional 412 patients followed under the auspices of the ProAR were classified as having mild to moderate asthma and were also included in the study. The inclusion criteria were being > 18 years of age and living in the city of Salvador, Brazil. The exclusion criterion was having other chronic or acute diseases of the lower airways or lungs. 

The present study was approved by the Research Ethics Committee of the Climério de Oliveira Maternity Hospital (Ruling no. 099/2010), located in the city of Salvador, Brazil. A subpopulation of asthma patients was recruited for gene expression assays, and this subproject was approved by the Research Ethics Committee of the Federal University of Bahia School of Medicine (Protocol no. CAAE 82928818.9.0000.5577/2.549.881). All participating patients gave written informed consent. 

Symptom control was assessed by the six-item Asthma Control Questionnaire, previously validated for use in Brazil.^17^ Scores of < 1.5 indicate controlled/partially controlled asthma, whereas scores ≥ 1.5 indicate uncontrolled asthma. All participating patients underwent spirometry before and 15 min after bronchodilator (albuterol) administration, as recommended by the American Thoracic Society/European Respiratory Society.^18^ A KoKo spirometer (KoKo PFT, Longmont, CO, USA) was used, with predicted values for the Brazilian population being used as reference; a 12% (and ≥ 200 mL) increase in FEV_1_ was considered significant for a β_2_ agonist response, as follows (BDR = 100 × ((post-FEV_1_ − pre-FEV_1_)/pre-FEV_1_)).^19^

We performed a retrospective longitudinal analysis of spirometry data from 309 severe asthma patients in the 18- to 88-year age bracket (2,349 observations, with intervals of 3-15 years [mean, 11 ± 2 years] and a mean of 8 BDR measurements per patient) to evaluate BDRs. This approach was used in order to capture temporal changes in BDR and assess the influence of genetic factors and aging. 

### Blood samples, genotyping, and gene expression

Peripheral blood samples were collected for DNA extraction in accordance with the manufacturer instructions (Gentra Puregene blood kit; QIAGEN, Hilden, Germany). Genotyping of the DNA samples was performed with the Infinium^®^ Multi-Ethnic AMR/AFR BeadChip microarray (Illumina, Inc., San Diego, CA, USA). Data for the *ADRB2* gene were extracted from the Genome Reference Consortium Human Build 37 (available at www.ncbi.nlm.nih.gov) with a ± 10,000 bp margin. 

Two SNPs with a minor allele frequency of < 5% and 64 individuals with < 90% genotyping rates were excluded, a total of 749 individuals remaining for association analyses. A subset of 31 individuals, selected by genotype, was used for functional studies, peripheral blood mononuclear cells being isolated with HisTopaque^®^-1077 (Sigma-Aldrich, Burlington, MA, USA). The Invitrogen™ PureLink™ RNA Mini Kit (Thermo Fisher Scientific, Waltham, MA, USA) was used for the extraction of RNA to be used in the expression assay, and reverse transcription was performed with the SuperScript™ IV First-Strand Synthesis System (Thermo Fisher Scientific). 

Real-time PCR was carried out on the QuantStudio 12K Flex Real-Time PCR System (Thermo Fisher Scientific) using *ADRB2* TaqMan^®^ Gene Expression Assays (Thermo Fisher Scientific), with *ACTB* as the normalization gene and non-risk genotypes serving as reference for relative expression calculations. 

### Platforms, databases, and statistical analysis

Genetic data for the SNPs were obtained from the U.S. National Center for Biotechnology Information SNP database (available at https://www.ncbi.nlm.nih.gov/snp/) and the Ensembl genome browser (available at https://www.ensembl.org/index.html), and SNPStats and PLINK 1.9 were used in order to assess the impact of multiple SNPs on the evaluated phenotypes.^20^ Linkage disequilibrium between markers was analyzed with Haploview, version 4.2.^21^

The IBM SPSS Statistics software package, version 22 (IBM Corporation, Armonk, NY, USA) was used for preliminary analyses, including assessment of covariates and variables through mean comparisons (by means of the Kruskal-Wallis test or ANOVA, depending on group distribution) and chi-square tests. Association analyses between phenotypes and variants were performed with PLINK 1.9 and R software, version 4.0.2 (The R Foundation for Statistical Computing, Vienna, Austria) under additive, recessive, and dominant models. Logistic regressions adjusted for sex, age, BMI, smoking exposure (primary, secondary, current, or former), and a major ancestry marker component were conducted; only SNP associations with p < 0.05 were considered significant, with permutation procedures used in order to reduce false-positive results.^22^

With the use of R software, version 4.0.2, linear mixed fixed- and random-effects models-with age, additive genotype effects, an age × genotype interaction, and subject-specific intercepts and slopes as random effects-were performed with the *lmerTest* package. Additionally, a generalized additive mixed model via the *gamm4* package was used in order to assess the impact of age on longitudinal BDR by genotype, with genotype-specific smooth and density plots created with *ggplot2*. All analyses were adjusted for sex, smoking exposure, BMI, and an ancestry marker. 

Centered smoothing plots were generated, and the 95% confidence intervals around the smoothed curves were depicted as shaded areas. Results were adjusted for covariates. R software packages were used in order to draw a correlation plot between BDR (BDR = 100 × ((post-FEV_1_ − pre-FEV_1_)/pre-FEV_1_)) and age (in years) in the study population. 

Other graphics and linear regression analyses, as well as expression analyses, were performed with GraphPad Prism, version 5 (GraphPad Software, Inc., San Diego, CA, USA), the Mann-Whitney test or the Student’s t-test being used depending on group distribution. 

## RESULTS

In the severe asthma group, there was a higher proportion of females, older individuals, smokers, and individuals with a higher BMI. All individuals with severe asthma regularly used ICS + LABAs (Table 1). 

**Table 1:** Characteristics of the study population.^a^

	Mild to moderate asthma (n = 383)	Severe asthma (n = 366)	p
Sex			0.02
*Female*	297 (77.5%)	297 (81.1%)	
*Male*	86 (22.5%)	69 (18.9%)	
Age, years	36 ± 13	51 ± 14	< 0.01
Exposure to smoking			< 0.01
*No*	278 (72.6%)	233 (63.7%)	
*Yes*	105 (27.4%)	133 (36.3%)	
BMI, kg/m^2^	26.99 ± 5.78	29.11 ± 5.60	< 0.01
Use of ICS + LABAs	0 (0.0%)	366 (100.0%)	< 0.01

ICS: inhaled corticosteroids; and LABAs: long-acting β_2_ agonists. ^a^Data expressed as n (%) or mean ± SD.

The *ADRB2* gene variants investigated in the present study were characterized in terms of the polymorphic allele (A1), the reference allele (A2), the potential functional role, and the minor allele frequency in the ProAR population (Table 2). The SNPs included in the analyses are located in coding regions of the *ADRB2* gene (Figure S1) and showed a low level of linkage disequilibrium (Figure S2). 

**Table 2:** Single nucleotide polymorphisms included in the analysis.

SNP	A1	A2	MAF	Function
rs1042713	A	G	0.44	Missense
rs1042714	C	G	0.24	Missense
rs1042717	A	G	0.32	Synonymous
rs1800888	A	G	0.01	Missense
rs3729943	G	C	0.01	Missense

SNP: single nucleotide polymorphism; A1: polymorphic allele; A2: reference allele; and MAF: minor allele frequency.

We found no significant associations of asthma severity with SNPs or sets of SNPs, a finding that suggests that there is no relationship between the genetic profile evaluated in the present study and the phenotype in question. None of the variants were associated with a lack of BDR in the cross-sectional analysis, even when the population was stratified by disease severity. 

The linear mixed fixed- and random-effects models revealed no significant associations between the main effects (age and genotype) or their interactions and BDR. In contrast, comparative analyses with generalized additive mixed models allowed us to visualize, through smoothing, significant trends in the longitudinal variation of BDR as a function of age by genotype. An independent analysis of genotype showed that BDR decreases over time (F = 11.79; p < 0.001; Figure S3). 

**Figure 1:**
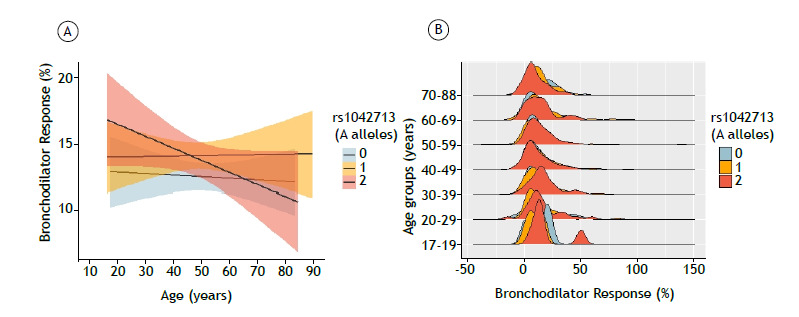
Correlation between rs1042713 and variations in longitudinal bronchodilator response. In A, overlaid smooths with a shaded area stratified by the number of A alleles-0, 1, or 2-and bronchodilator response adjusted for covariates. In B, density graphs for individuals stratified by bronchodilator response, grouped by number of A alleles and age.

**Figure 2:**
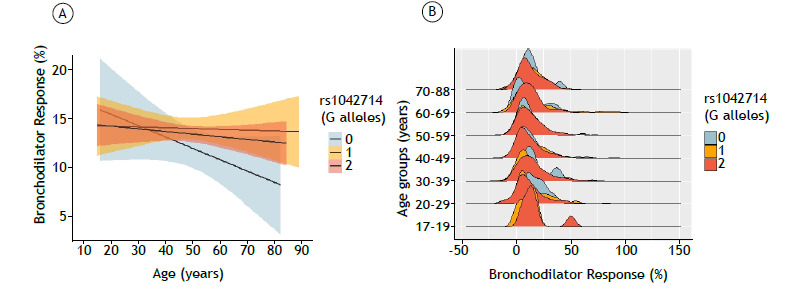
Correlation between rs1042714 and variations in longitudinal bronchodilator response. In A, overlaid smooths with a shaded area stratified by the number of G alleles-0, 1, or 2-and bronchodilator response adjusted for covariates. In B, density graphs for individuals stratified by bronchodilator response, grouped by number of A alleles and age.

The presence of the A (A46) allele of rs1042713 in heterozygous form (F = 4.16; p = 0.04) and in homozygous form (F = 2.98; p = 0.03) was related to a greater variation in acute BDR with age (Figure 1). The presence of the reference G (G79) allele of rs1042714 in homozygous form characterized the absence of C79 polymorphic alleles (F = 8.56; p < 0.01; Figure 2). The reference G (G252) allele of rs1042717 in homozygous form (F = 8.28; p < 0.01) and heterozygous form (F = 4.26; p = 0.04) was also related to a greater variation in BDR with age in comparison with the standard response (Figure S4). 

A joint longitudinal analysis of the rs1042713 and rs1042714 variants showed that individuals with four risk alleles (A46A46-G79G79) had a more pronounced variation in acute BDR with increasing age (F = 3.01; p = 0.03; data not shown). The other combinations of risk alleles showed no significant results. 

In recessive models, the G (G79) allele of rs1042714 was found to be associated with severe uncontrolled asthma (OR = 1.75; permutation p = 0.02). Similarly, the A (A252) allele of rs1042717 showed an association with a lack of disease control in patients with severe asthma (OR = 2.06; permutation p = 0.04). No other SNP in the *ADRB2* gene was associated with asthma control in any genetic model for individuals with severe asthma (p > 0.05; Table 3). Our allelic analysis showed that a specific combination of genotypes formed by the evaluated SNPs was associated with a lack of disease control in individuals with severe asthma. The set containing the G (G46) allele of rs1042713, the G (G79) allele of rs1042714, and the A (A252) allele of rs1042717 showed a different outcome in comparison with that in individuals with other genotypic combinations (OR = 3.2; p = 0.02). In addition, the presence of one of the genotype sets formed among the evaluated SNPs was associated with a lack of disease control in individuals with severe asthma. The set of genotypes containing the G (G46) allele of rs1042713, the G (G79) allele of rs1042714, and the A (A252) allele of rs1042717 showed a significantly different outcome when compared with that in individuals with other sets of genotypes (OR = 3.2; p = 0.02; Table 4). No other combination of alleles was found to be statistically significant. The genotypes of rs1042713, rs1042714, and rs1042717 did not affect the relative quantification of *ADRB2* gene expression between the groups of patients with severe asthma (Figure S5). 

**Table 3:** Associations of rs1042714 and rs1042717 with asthma control in individuals with severe asthma.

Genotype	Severe controlled/partially controlled asthma - n (%)	Severe uncontrolled asthma - n (%)	OR (95% CI) Permutation p*
rs1042714			
CC/CG	115 (46.6%)	38 (32.8%)	1.75 (1.10-2.86) Permutation p = 0.02
GG	132 (53.4%)	78 (67.2%)	
rs1042717			
AG/GG	224 (90.1%)	96 (82.8%)	2.06 (1.06-4.01) Permutation p = 0.04
AA	23 (9.9%)	20 (17.2%)	

A: adenine; C: cytosine; and G: guanine. *Only significant results (permutation p < 0.05) are presented.

**Table 4:** Associations of sets of rs1042713 (G46), rs1042714 (G79), and rs1042717 (A252) genotypes of the *ADRB2* gene with severe asthma control.

Allele set	Severe controlled/partially controlled asthma - n (%)	Severe uncontrolled asthma - n (%)	OR (95% CI)	p*
Ref.	107 (43.3%)	39 (33.7%)	3.2 (1.21-8.47)	0.02
G46/G79/A252	140 (56.7%)	77 (66.3%)		

*Only significant results (p < 0.05) are presented.

## DISCUSSION

Missense variants of the *ADRB2* gene, such as rs1042713 (G46A) and rs1042714 (G79C), which promote amino acid changes and affect receptor structure and function,^23^ ^,^ ^24^ were associated with SNP rs1042717 and genotype sets linked to asthma control and a lack of acute response to short-acting bronchodilators in severe asthma patients evaluated longitudinally. However, we cannot determine whether these variants alter gene expression, given that exon variants are less often implicated in regulatory effects than are non-coding ones.^25^ ^,^ ^26^ This finding suggests that the observed clinical impact is likely due to structural changes in the receptor rather than to alterations in gene expression, indicating that the effect of these variants occurs through a mechanism other than transcriptional regulation. 

No significant differences in acute BDR were found among patients with asthma. Importantly, all individuals diagnosed with severe asthma were using continuous LABAs. It is known that chronic exposure to β_2_ agonists results in downregulation of β_2_ receptors,^9^ ^,^ ^15^ with reduced responsiveness and consequent tachyphylaxis to LABAs.^15^ Our finding corroborates those of a meta-analysis^27^ in which the authors analyzed five studies of the A46G polymorphism and concluded that it was not associated with BDR in asthma patients who chronically used ICS + LABAs.^27^ Furthermore, it is known that age is an important modifying factor of genetic predisposition. 

In our longitudinal analysis, we identified correlations between different genotypes and changes in BDR over time. Unlike what we found in our cross-sectional analysis, our longitudinal analysis showed a significant implication of the A46 allele in BDR variation with age. This result may indicate a possible reversal of the effect of that allele on asthma patients, given that the results indicate greater reversibility at ages closer to 18 years, different from what was found in older individuals, in whom reversibility levels were lowest among the different genotypes of rs1042713. This inversion of effects might explain why cross-sectional analyses failed to detect significant associations with that SNP. 

One cohort study showed that individuals homozygous for A46 using LABAs regularly experienced a marked reduction in BDR in comparison with A46 homozygotes using SABAs sporadically and individuals homozygous for G46 using LABAs.^15^ Furthermore, the A46 allele was associated with tachyphylaxis,^28^ supporting the notion that A46 isoforms undergo increased downregulation and decreased cyclic adenosine 3’,5’-monophosphate response after repeated bronchodilator use, suggesting a link between that allele, receptor efficiency, and alterations in the lysosomal degradation process of the β2-adrenergic receptor.^9^

We found that individuals with the G79 allele show a stable BDR over time, whereas those with the C79C79 genotype show a reduced response; this finding is consistent with those of an in vitro study showing that the G79G79 genotype confers greater resistance to downregulation following prolonged β_2_ agonist stimulation in fibroblast cultures.^29^ In a case-control study, the presence of the C79 allele was linked to tachyphylaxis, whereas the G79 allele acted as a protective factor, promoting a favorable BDR.^28^

We also analyzed the impact of different sets of rs1042713 and rs1042714 genotypes on the reversibility of asthma with age. The results corroborated other findings of ours, indicating that individuals with the A46A46-G79G79 genotype show a greater change in BDR with age in comparison with those with other genotypes (data not shown). 

We then evaluated the influence of these genetic variants on severe asthma control under regular therapy. Our results indicate that the G79G79 and A252A252 genotypes correlate with poor control despite treatment with LABAs and ICS, whereas rs1042713 showed no association with a lack of asthma control. These findings are consistent with those of previous studies using the six-item Asthma Control Questionnaire ^30^ and studies involving pediatric populations,^31^ although Sood et al.^31^ reported an association of rs1042714 with exacerbation risk via the C allele, a discrepancy that may be partly explained by population stratification. 

In order to assess the joint effect of rs1042713, rs1042714, and rs1042717, we analyzed all possible genotypic combinations. We found that the presence of the G46 and G79 alleles (in block with A252) had a greater impact on severe asthma control than did the individual genotypes. Collectively, these results demonstrate that these variants influence respiratory disease monitoring, given that spirometry, which is used for asthma diagnosis, severity assessment, and treatment effectiveness assessment, is based on the individual response to short-acting β_2_ agonists, a response that has been shown to be affected by these variants.^32^ ^,^ ^33^

It is important to consider that the lack of individuals with three or more polymorphic alleles in our population represents a limitation of our study, given that these data could better characterize all the possibilities of genotypic combinations among the studied variants. It was also not possible to recruit a larger number of individuals for gene expression analysis. The present study did not assess the effects of socioeconomic factors,^34^ housing,^35^ working conditions,^36^ the airway microbiome,^37^ epigenetic data,^38^ or other factors that have been shown to modify asthma phenotypes.^39^ ^,^ ^40^

In summary, the rs1042713, rs1042714, and rs1042717 variants are associated with BDR changes with age in asthma patients, and asthma control is significantly linked to these SNPs; furthermore, continued LABA exposure may interfere with the acute response to β_2_-adrenergic drugs, suggesting that chronic bronchodilator use may modify the genetic effect. These findings enhance our understanding of the mechanisms behind β_2_ agonist-induced bronchodilation and its impact on spirometry, paving the way for personalized asthma therapy based on individual genetic profiles. Although challenges such as cost and accessibility persist, the continuous improvement of a genetic panel shows promising potential. 
